# Federated Learning with Research Prototypes: Application to Multi-Center MRI-based Detection of Prostate Cancer with Diverse Histopathology

**DOI:** 10.1016/j.acra.2023.02.012

**Published:** 2023-03-12

**Authors:** Abhejit Rajagopal, Ekaterina Redekop, Anil Kemisetti, Rushikesh Kulkarni, Steven Raman, Karthik Sarma, Kirti Magudia, Corey W. Arnold, Peder E.Z. Larson

**Affiliations:** Department of Radiology and Biomedical Imaging, University of California, San Francisco, 94158, USA (A.R., A.K., P.E.Z.L.); Departments of Radiology and Electrical Engineering, University of California, Los Angeles, 90024, USA (E.R., R.K., S.R., K.S., C.W.A.); Department of Radiology, Duke University, Durham, 27708, USA (K.M.)

**Keywords:** Federated learning, Prostate MRI, Deep learning, Cancer classification, Gleason scores

## Abstract

**Rationale and Objectives.:**

Early prostate cancer detection and staging from MRI is extremely challenging for both radiologists and deep learning algorithms, but the potential to learn from large and diverse datasets remains a promising avenue to increase their performance within and across institutions. To enable this for prototype-stage algorithms, where the majority of existing research remains, we introduce a flexible federated learning framework for cross-site training, validation, and evaluation of custom deep learning prostate cancer detection algorithms.

**Materials and Methods.:**

We introduce an abstraction of prostate cancer groundtruth that represents diverse annotation and histopathology data. We maximize use of this groundtruth if and when they are available using UCNet, a custom 3D UNet that enables simultaneous supervision of pixel-wise, region-wise, and gland-wise classification. We leverage these modules to perform cross-site federated training using 1400+ heterogeneous multi-parameteric prostate MRI exams from two University hospitals.

**Results.:**

We observe a positive result, with significant improvements in cross-site generalization performance with negligible intra-site performance degradation for both lesion segmentation and per-lesion binary classification of clinically-significant prostate cancer. Cross-site lesion segmentation performance intersection-over-union (IoU) improved by 100%, while cross-site lesion classification performance overall accuracy improved by 9.5-14.8%, depending on the optimal checkpoint selected by each site.

**Conclusion.:**

Federated learning can improve the generalization performance of prostate cancer detection models across institutions while protecting patient health information and institution-specific code and data. However, even more data and participating institutions are likely required to improve the absolute performance of prostate cancer classification models. To enable adoption of federated learning with limited re-engineering of federated components, we open-source our FLtools system at https://federated.ucsf.edu, including examples that can be easily adapted to other medical imaging deep learning projects.

## INTRODUCTION

Prostate cancer is the most prevalent cancer in American men but data shows that it affords a 99% survival rate if the cancer is detected early (1). Compared with the low specificity of PSA blood tests and the potential complications of invasive biopsy, screening based on magnetic resonance imaging (MRI) offers the potential of a fast, safe (non-invasive), and localized detection of prostate cancer that can aid in both diagnosis and treatment planning (2). Although the PI-RADS version 2.0 introduced much needed standardization of prostate MRI reporting (3), the scores associated with individual lesions has been shown to correlate poorly with the Gleason grade (cancer severity) determined by biopsy and histopathological analysis, with a positive predictive value of just 35% (4,5). While the introduction of PI-RADS version 2.1 aimed to improve this reporting system (6), the changes have been shown to have limited effect in improving overall cancer detection rates due to the changes only taking effect in a very small number of patients (7). As such, there is great interest in developing data-driven algorithms to assist and improve radiologists’ capabilities in detecting and staging of clinically-significant prostate cancer (CS-PCa).

Unfortunately, accurate and early MRI-based detection of prostate cancer has eluded machine learning and even deep learning algorithms to-date. While positive results have shown improved accuracy in staging of clinically-significant disease (Gleason patterns >3 + 3, Gleason grade group ≥2) (8), the overwhelming majority of deep learning prostate cancer detection studies have found similar accuracy to PI-RADS when utilizing screening populations with a balanced representation of Gleason grade groups (9). Such models still have clinical value, e.g. for transferring knowledge from expert radiologists, but requires models to have strong generalization behavior both within and across institutions, where MRI protocols, granularity of biopsy data, MRI hardware, and patient populations can vary significantly (10,11). This is especially important for application to screening populations where prostate cancer may present subtly, and single-site training may suffer from inadvertent bias or brittleness that ultimately limits performance even on data collected at the same institution.

Federated learning (FL) presents an opportunity here to overcome these barriers, by offering a platform to share models and abundant data for extensive cross-site validation and enhanced model training without the need to share images or other sensitive protected health information (PHI). However, existing FL implementations have many limitations, such as the use of a limited set of only the most common neural network architectures, freezing of model code and data pipelines, intra- and inter-institution data homogeneity, or release of institution-specific code or data, which creates barriers for inclusion of research sites and limits the use of specialized approaches (12). This is especially prohibitive for medical imaging problems where patient examination protocols differ by research site, deep learning is still exploratory, and the community has not agreed on an ideal model architecture or data format, as is true for MRI-based assessment of prostate cancer.

**Fig. 1.**
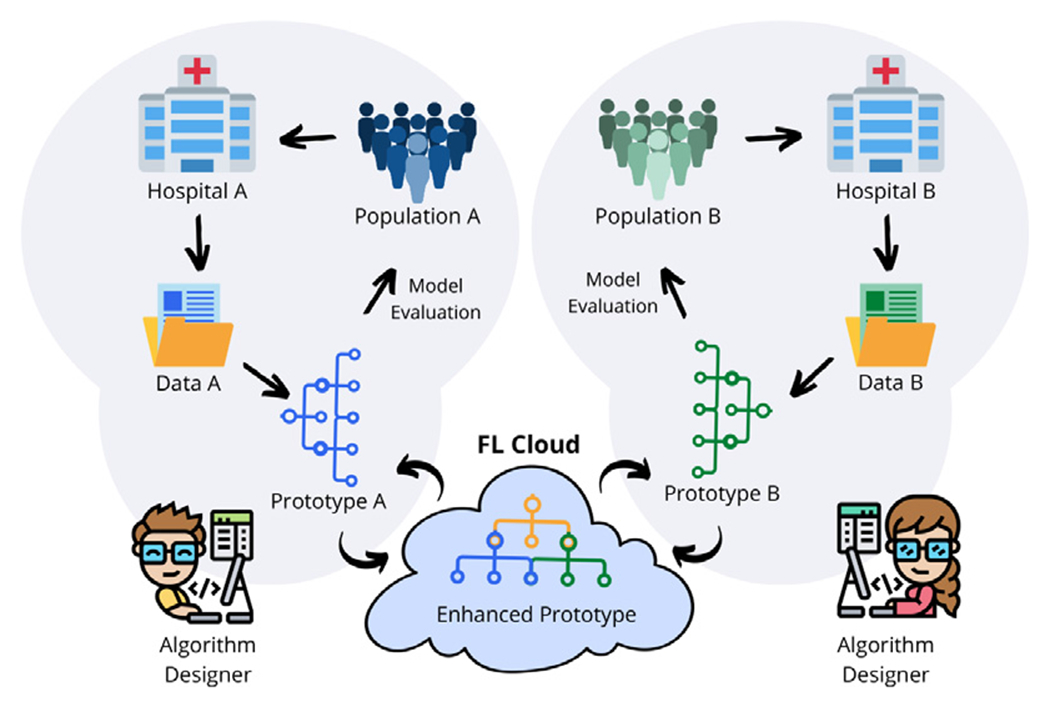
Federated learning of research prototype algorithms enables cross-site validation that is useful for improving within-site performance. (Color version of figure is available online.)

Instead, what is needed is a flexible environment to apply deep learning models at scale, while retaining the ability to debug operations, modify model hyperparameters and data pipelines, and handle heterogeneous data formats, while maintaining security, privacy, and *autonomy* of code and data from participating institutions (Figure 1). Federated learning, in its truest sense, implies that each institution should be free to maintain separate implementations, as long as they adhere to the rules of federation, or in this case an application programming interface (API). To this end, in this paper we introduce a design pattern for federated learning of research prototypes, that provides a template API for FL with the following high-level features:

- *Private institution-specific code and data stays private*. Each institution does not need to share dataloading, pre-processing, or training code or with other institutions, or even the central server. For example, gradients or weights are shared via an API call, so institution-specific implementations are never revealed.
- *All federated components and model code are public domain*. This allows each institution to verify security, privacy, and operation of the FL system. Our threat model treats each institution as a trusted party, with the central server being honest but curious, and communication only over secure channels. Further, each client has programmatic control over what is sent to the central server, preventing erosions of contract.
- *Custom models, training strategies, and federated aggregation are left to researchers*. The sole requirement is for each client to provide a get_objects function that assembles objects for local training. The distributed training strategy (implemented in the backend using NVFlare (13), or similarly using Flower (14) is built on top of the local training objects–not code, so researchers are free to experiment with new models and strategies without modifying any federated components.

We concertize this design pattern in an open-source toolkit, FLtools, which provides the necessary model- and data-agnostic federated components to train any feed-forward deep neural network using federated learning using, with *zero* modifications to site-specific model architecture code or local training routines. We achieve this by defining data and model abstractions that are used by both local- and federated-training routines, but which can crucially differ across federated clients and research sites. Our toolkit is freely available at https://federated.ucsf.edu, and includes a simulation system, FLsim, that allows for model debuging and inspection of intermediate tensors while applying the same routines used in the full-scale FL system.

As an illustrative example, in this paper we use FLtools to perform cross-institution training of a custom deep learning model for multi-parametric MRI-based detection and classification of prostate cancer. For this task, we the recently proposed UCNet model architecture (15), which features a fully-convolutional 3D UNet backbone with a 3D region-of-interest (ROI) classification head that enables per-lesion, per-sextant, and ultimately per-exam prediction of prostate cancer severity. The UCNet model achieves this by utilizing a histogram representation of the International Society of Urological Pathology (ISUP) grade group (or Gleason grade group) (4) that reflects a common data representation for prostate cancer histopathology across research sites, which may themselves include exams and groundtruth histopathology data at various granularities. The flexible UCNet, combined with the proposed federated learning system, enables the use of herterogeneous exams with groundtruth data from targeted biopsy, systematic biopsy, and prostatectomy, as well as results reported at a lesion, sextant and whole gland level.

### Prior Work

Prior work in federated learning (FL) includes both problems of theoretical interest (learning strategies under various constraints, such as communication bottlenecks, non-iid data, or privacy concerns) and of practical value; with the latter focusing on the development of tools and frameworks to alleviate various user constraints. McMahan et al. provides an excellent overview of various available FL frameworks and research problems (16). One of the primary practical challenges an FL system faces is making the workflow as straightforward as possible, ideally approaching the ease-of-use achieved by machine learning libraries for local (single computer) training. In this paper we address this pain point by developing a library and design pattern that extends NVidia’s NVFlare toolkit for federated training using *reusable, replacable*, and *interoperable* federated components.

Specifically for prostate cancer detection from MRI, several works have utilized federated learning. Sarma et al (11) used multi-center federated training improves prostate gland segmentation, an important sub-step in the search for prostate cancer biomarkers. Yan et al (17) proposed a variation-aware federated learning framework where where the variations among clients are minimized by transforming the images (ADC maps) of all clients onto a common image space. Although some degree of homogeneity of input MRI (sequences, resolution, contrast normalization) is required for detection models to fully-leverage big datasets, traditional approaches to FL (including in (11) and (17) homogenize *annotation datatypes*, which is an important discrete variation both within and across institutions. In contrast, our work addresses this variation directly with a model architecture that leverages highly heterogeneous radiological annotation and histopathology. We evaluate the efficacy of this approach using multi-center federated learning with large datasets collected at UCSF and UCLA research hospitals.

## MATERIALS AND METHODS

### Dataset and Assumptions

#### IRB Approval

The data and analysis for this study was approved by UCSF and UCLA Institutional Review Boards (IRB).

#### Multi-parametric Magnetic Resonance Imaging

Prostate MRI protocols are typically multi-parametric acquisitions composed of T2-weighted images (T2WI), diffusion-weighted images (DWI), and dynamic contrast-enhanced (DCE) MRI. T2WI MRI provides anatomic data, DWI and associated apparent diffusion coefficient (ADC) maps represent the restriction of water movement in tissue that is altered in prostate tumors compared to healthy tissue, and DCE assesses differences in vascularity and blood supply. PI-RADS primarily relies on DWI and T2WI with a limited role and influence from DCE in select cases. An abbreviated biparametric (bp-MRI) exam involving just T2WI and DWI has been increasingly explored given the limited role of DCE in PI-RADS as well as the added time, complexity, cost, and contrast agent risks it requires. Herein, we investigate the use of bp-MRI for prostate cancer classification. Specifically, the T2-weighted and DWI as well as the associated ADC maps were extracted from a mp-MRI exam, and used in all the subsequent analysis.

All MRI data was acquired on 3T MRI scanners. At UCSF, the scans were performed on MRI scanners from GE Healthcare, and primarily using an endo-rectal receive RF coil. At UCLA, the scans were performed on MRI scanners from Siemens Healthineers typically with external receive RF coil arrays (<2% endo-rectal coil). The typical parameters for the T2WI for UCSF were a 2D fast spin-echo sequence with TE = 102 ms, TR = 6 s, spatial resolution = [0.35, 0.35, 3] mm. The typical parameters for the T2WI for UCLA were a 3D turbo spin-echo sequence, TE = 201 ms, TR = 2200 ms, spatial resolution = [0.66, 0.66, 1.5] mm. The typical parameters for DWI for UCSF were TE = 50-65 ms, TR = 4-5 s, b = 600 s/mm^2^, and spatial resolution = [1.6, 1.6, 3] mm, or TE = 60-85 ms, TR = 4-5 s, b= 1350 s/mm^2^, and spatial resolution = [2, 2, 3] mm. The typical parameters for DWI for UCLA were TE = 80 ms, TR = 4.8 s, spatial resolution = [1.625, 1.625, 3.59] mm, b-value = 1400 s/mm^2^. All cases utilized in this study utilized high b-value DWI. All images were downsampled and/or interpolated to the same spatial resolution of [0.66, 0.66, 2.24] mm for x, y, and z axis respectively for all series in datasets from both participating research sites.

#### Gland Segmentation and Contrast Normalization

As T2 and DWI have arbitrary non-quantitative image amplitudes, we apply interquartile range (IQR_99_)-based intra-image normalization to address the relative nature of MR image intensity values both within and across research sites and to eliminate outlying values created by imaging artifacts. Specifically, each image was normalized to the image-level IQR_99_ computed within the 3D prostate gland (annotated by a radiologist or previously developed neural network segmentation model (18,19) as in (20):

(1) $$I_{\mathrm{norm}}=\frac{I-\text{percentile}(I,1)}{\text{percentile}(I,99)-\text{percentile}(I,1)}$$

**Fig. 2.**
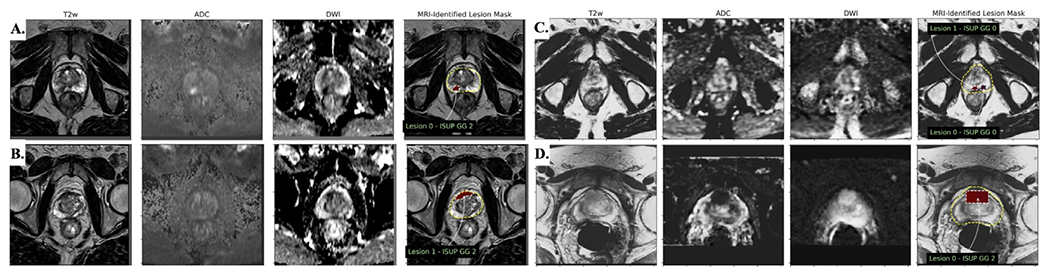
Intra- and inter-site variations of multiparametric MRI data. (A,B) Natural variation between appearance of ISUP grade group 2 lesions (countoured) in UCLA data. (C,D) Large variation in the apparent MR contrast and size of lesion annotations (bounding boxes) in UCSF data. (Color version of figure is available online.)

To overcome the problem of high variability of intensity distribution across patients, we additionally apply Z-score image normalization to transform T2WI and DWI image intensities within the prostate gland to have zero mean and unit variance. Although there remain differences in image characteristics between the datasets used at University A and University B even after this pre-processing (Figure 2), we find that providing sample-level normalization can significantly improve the performance of trained models. With additional coordination, significant improvements could be made by further homogenizing the input data using cross-sample and cross-institutional dataset normalization.

#### Histopathology Derived from Prostate Biopsy

Prostate MRIs are typically conducted after an indication of an elevated prostate-specific antigen (PSA) blood concentration. At both research sites, a board-certified radiologist annotated the mp-MRI series with possible lesions, suggesting areas for prostate biopsy. At UCSF, prostate biopsy is conducted by transrectal-ultrasound (TRUS) MR-guidance, where a T2 MRI series is fused with the ultrasound to help navigate the needle and target the MR-annotated regions. In addition to targeted biopsy, UCSF urologists systematically sample the prostate in 6 regions, providing additional confidence to the gland-wise cancer designation. Unfortunately, as the prostate is highly non-rigid, only coarse coordinates are associated with each biopsy core sample. That is, for UCSF data we assume the location of the systematic biopsies based on a geometric division of the prostate into sextants in the registered MR-image space, and we assume the targeted biopsy occurs within a 2D bounding box around each lesion in each slice of the registered MRI. The groundtruth histopathology for each biopsy sample was determined by a pathologist who observed stained slices of the biopsy core under a microscope and assigned a Gleason pattern to each. We convert this Gleason pattern to the standardized ISUP grade group (1-5, 0 for negative), where a grade group ≥2 indicates clinically significant prostate cancer (CS-PCa).

At UCLA, a similar targeted TRUS prostate biopsy is conducted, but with additional innovation to identify the location of needle in the joint ultrasound-MRI image space, and enhanced radiologist-defined contouring of each lesion. The histopathology for each biopsy core is determined in a similar fashion as UCSF, resulting in a set of ISUP grade group scores. In this paper however, for data from the UCLA site we assume the highest ISUP grade group in each exam is known, but not to which lesion they correspond. For the UCLA site, we also do not include any systematic biopsy data. This mismatch in available data provides additional consideration for the design and supervision of our chosen deep architecture and supervision system, as will become apparent in Section 2.2.

#### Summary of Datasets at Research Sites

Table 1 summarizes the distribution of ISUP grade groups in training, validation, and testing exams for both research sites. As each prostate gland may yield multiple biopsy cores, exams are grouped by the highest ISUP grade. As evident, our datasets retrain some imbalance with respect to the cancer grade group distribution, in addition to less tabulatable differences in image characteristics.

The demographic distribution with respect to the age, race, PSA density of these datasets is displayed in Table 2. Notably, these datasets reflect diverse screening and active surveillance populations, but are significantly imbalanced towards representing White men, due to both health equity and the populations served by UCSF and UCLA. We hope that the federated learning system proposed herein will enable the use of more diverse cohorts by breaking traditional geographic barriers of data sharing.

### UCNet for Mixed Histopathology Supervision

**Fig. 3.**
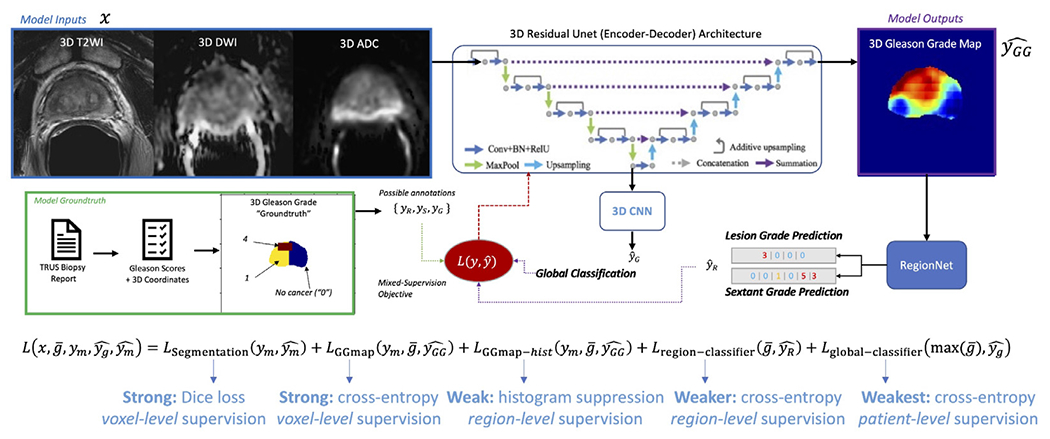
UCNet Architecture, depicted here with a 3D residual UNet backbone, histopathology-based histogram suppression, and regional classification modules. In this paper, UCNet takes registered 3D mp-MRI as input and produces as output: lesion segmentation maps, 1-hot-encoded cancer grading maps (for classification of clinically-significant prostate cancer, *K* = 2), and per-region classifications $\left({ℒ}_{\text{global}}\right.$ not trained). (Color version of figure is available online.)

For this problem, we utilize the recently proposed 3D UCNet model, which is essentially composed of a 3D residual UNet with an additional fully-connected classification output head (15). Our implementation (Figure 3) takes registered 3-channel bp-MRI as input and predicts 3D lesion segmentation maps, 3D ISUP grade group maps, and region-wise histograms that are used to determine region-wise and exam-wise cancer severity. Although UNet-like architectures are applicable to many deep learning tasks in medical imaging (11,19,21,22), UCNet has been shown to be particularly well-suited to jointly handling a variety of complementary groundtruth histopathology datatypes available for prostate cancer, albeit from a single institution (15).

Of central importance to the federated learning approach used herein is the dynamically-populated multi-task objective that we use to train UCNet with highly heterogeneous data collected both within and *across* institutions:

(2) $$ℒ\left(x,{𝒴}_{\text{seg}},{𝒴}_{\text{gg}},z\right)=\alpha_{1}\lambda_{1}{ℒ}_{\text{region–classifier}}+\alpha_{2}\lambda_{2}{ℒ}_{\text{GGmap–hist}}+\alpha_{3}\lambda_{3}{ℒ}_{\text{GGmap}}+\alpha_{4}\lambda_{4}{ℒ}_{\text{segmentation}}$$

where $x\in {\mathbb{R}}^{3\times X\times Y\times Z}$ represents the registered 3-channel (T2WI, DWI, ADC maps) input MRI, ${𝒴}_{\text{seg}}\in {\mathbb{R}}^{X\times Y\times Z}$ represents the groundtruth lesion segmentation mask, ${𝒴}_{\text{gg}}\in \mathbb{R}$ represents the groundtruth Gleason grade group for each of the *R* regions in an exam, and $z\in {\mathbb{R}}^{R}$ represents the type of supervision (1-strong or 2-weak) applicable to the groundtruth datatype (lesion-biopsy or systematic-biopsy) available for each region r∈R.

Specifically, the UCNet model uses x to predict a tanh-activated 3D lesion segmentation map ${\overset{ˆ}{𝒴}}_{\text{seg}}\in {\mathbb{R}}^{1\times X\times Y\times Z}$ and softmax-activated 3D cancer grade group map ${\overset{ˆ}{𝒴}}_{\text{gg}}\in {\mathbb{R}}^{2\times X\times Y\times Z}$, used to populate the objective terms for:

- lesion segmenatation, implemented using the combination:
(3) $${ℒ}_{\text{segmentation}}\left({𝒴}_{\text{seg}},{\overset{ˆ}{𝒴}}_{\text{seg}}\right)={ℒ}_{\text{seg–dice}}+{ℒ}_{\text{seg–BCE}}$$
  where
(4) $$\frac{{ℒ}_{\text{seg–dice}}\left({𝒴}_{\text{seg}},{\overset{ˆ}{𝒴}}_{\text{seg}}\right)=1-{𝒴}_{\text{seg}}\cdot {\overset{ˆ}{𝒴}}_{\text{seg}}}{{𝒴}_{\text{seg}}+{\overset{ˆ}{𝒴}}_{\text{seg}}+\epsilon }$$
- strong voxel-wise cancer grading in homogeneous lesions, implemented using the standard categorical cross-entropy:
(5) $${ℒ}_{\text{GGmap}}\left(z,{𝒴}_{\text{gg}},{\hat{𝒴}}_{\text{gg}}\right)=\frac{1}{\left|R^{+}\right|}\;\sum_{r\in R^{+}}\sum_{k=1}^{K}{𝒴}_{\text{gg}}[r,k]\text{log}{\hat{𝒴}}_{\text{gg}}{\left[1_{r}\right]}_{k}$$
  where 1_*r*_, represents an indicator (Kronecker delta) function selecting the voxels corresponding spatially to region r, and herein we pick K=2 to represent the binary detection problem for CS-PCa.
- and, weak spatial cancer grading in gland regions with heterogeneous tissue types, implemented as ${ℒ}_{\text{GGmap–hist}}={ℒ}_{\text{hist–strong}}+{ℒ}_{\text{hist–high}}$ via $\overset{ˆ}{h}$, the soft K-dimensional histogram of predictions in each region, where:
$${ℒ}_{\text{hist–strong}}\left(z,{𝒴}_{\text{gg}},\hat{h}\right)=\frac{1}{\left|R^{\alpha }\right|}\;\sum_{r\in R^{\alpha }}\sum_{k=1}^{K}{𝒴}_{\text{gg}}[r,k]\text{log}\hat{h}[r,k]$$
  represents the sum over the conventional categorical cross-entropy of each histogram bin in each region $r\in R^{\alpha }$ where the supervision signal z[r] is 1 (lesion biopsies), and
$${ℒ}_{\text{hist–high}}\left(z,{𝒴}_{\text{gg}},\hat{h}\right)=\frac{1}{\left|R^{\beta }\right|}\;\sum_{r\in R^{\beta }}\;\sum_{k>{\text{argmax}}_{k}{𝒴}_{\text{gg}}[r]}^{K}{𝒴}_{\text{gg}}[r,k]\text{log}\hat{h}[r,k]$$
  represents the modified sum over the categorical cross-entropy of each histogram bin *greater* than the groundtruth biopsy score, in each region $r\in R^{\beta }$ where the supervision signal z[r] is 2 (systematic biopsies). The idea of ${ℒ}_{\text{GGmap–hist}}$ is to suppress the proportion of voxels representing grade groups not supported by the histopathology data, namely for hetereogeneous tissue where the histogram bin corresponding to the groundtruth grade group may not have the highest proportion.

Finally, the histograms $\overset{ˆ}{h}$ derived from the predictions ${\overset{ˆ}{𝒴}}_{\text{gg}}\in {\mathbb{R}}^{K\times X\times Y\times Z}$ are fed to the RegionNet classification head to produce region-wise predictions ${\overset{ˆ}{𝒴}}_{\text{gg–region}}$, that are supervised with the standard cross-entropy objective:

$${ℒ}_{\text{region–classifier}}\left(z,{𝒴}_{\text{gg}},\hat{z}\right)=\frac{1}{\left|R^{\text{*}}\right|}\;\sum_{r\in R^{\text{*}}}\sum_{k=1}^{K}{𝒴}_{\text{gg}}[r,k]\text{log}\hat{z}[r,k]$$

Additional details on the UCNet approach and baseline experiments with comparisons to state of the art are covered comprehensively in (15).

### Modular Framework for Federated Learning

We are using federated learning (FL) as a way to combine and maximize the use of prostate MRI exams across research sites with minimal impact to research prototype algorithm design and existing training and validation pipelines. Although the chosen UCNet model provides considerable flexibility for handling diverse histopathology datatypes associated with prostate MRI, the goal herein is to develop an approach for handling diversity in datatypes *across* institutions regardless of the specific model chosen.

Distributed FL typically requires adapting a “local” (single-computer) training loop into a “federated” training loop, where model weight updates are synchronized between the remote clients and a central server to implement learning algorithms like federated stochastic gradient descent (FedSGD) or weight averaging (FedAverage). Typically this synchronization demands advanced engineering and researchers consequently often rely on FL frameworks to implement the details and help them build federated loops quicker.

To achieve this, many frameworks (e.g. NVidia Clara, Flower) use programming design patterns like dependency injection to maintain control of the main program logic (e.g. looping structures) while also providing programming hooks for custom code. Although this simplifies the interface for federated deployment and increases adoption for well-calibrated production-ready models in established problem domains, this presents an obstacle for researchers developing new models in problem domains at their infancy, such as prostate cancer detection from MRI. This is because the researcher cannot easily develop, debug, and refactor the core model or training logic as a module independent of the FL framework engineering code. For example, even hardware-agnostic frameworks like Flower (14) require re-implementation of the federated loop.

To address this, we present a design pattern for FL that separates model development and FL implementation code, providing a feature-rich FL development environment for medical imaging researchers. Our design pattern is composed of 3 elements: a model abstraction (Section 2.3.1), a data abstraction (Section 2.3.1), and a model-agnostic federated toolkit (Section 2.3.1). The model and data abstractions are extensible and are meant to expose essential functionality of local gradient computation and weight updating to the federated toolkit without requiring any re-implementation. The federated toolkit can be implemented with different distributed frameworks on the backend, such as with Nvidia’s NVFlare 2.1 or Flower. In the current implementation our federated toolkit, FLtools seamlessly handles any differentiable feed-forward network architecture implemented in torch.

#### Model Abstraction

We adopt the pytorch lightning (23) design pattern for specifying models, which involves defining a class with the key methods:

- forward – a function that implements a forward pass or inference given a batch of *input* data, using the model architecture and weights (objects defined in the class), resulting in a set of tensors as the model’s *output*.
- training_step – a function that accepts a batch of training data (paired *input* data and *groundtruth* data), calls forward with *input* data to produce *output* data, and computes *metrics* and *losses* by comparison with the *groundtruth* to both monitor and train the model.
- validation_step – (optional) a function that performs the same function on training_step using a frozen version of the model on a batch of validation data, to monitor model performance.
- unpack_batch – (optional) a function we define to accept and unpack a dictionary of collated training or validation data, to enable easy dispatch of forward and training_step without complex arguments.
- configure_optimizers – a function that returns a neural network optimization function of the user’s choice.

Our current implementation utilizes torch networks encapsulated within a pytorch lightning class, but a similar design pattern is equally applicable to other deep learning frameworks like tensorflow. One distinct benefit of utilizing a pytorch lightning class is that existing local logging and checkpointing functionality can be utilized during federated training as well.

#### Data Abstraction

There are two abstractions we use here, one for the general FL design pattern, and the second for the specific MRI-based prostate cancer localization and classification problem.

For the general FL design pattern, we require each client to provide a get_objects function that returns the following:

- model – a python class, defined with the aforementioned member functions and learnable algorithm parameters (Section 2.3.1).
- train_dataloader – a python iterable that (when iterated on) returns a collated dictionary of batched training data using client-specific code and data sources. This can be defined using private, client-specific file loaders and a torch.utils.data.DataLoader to perform collation.
- val_dataloader – same as train_dataloader, but returning a collated dictionary of validation data.

Thus, from the perspective of the FL training loops, data yielded by client-specific dataloaders are fed directly to training_step, without additional specification or connector code within the FL environment. This provides flexibility for prototype algorithm developers at each research site to change model and data specifications (e.g DNN toplogy, file loading, pre-processing, augmentation) without modifying the interface. Moreover, each site is free to modify and optimize local implementations of all the aforementioned components (including choice of training objectives and metrics), albeit within constrains of the federated algorithms employed.

Specifically, for the MRI-based prostate cancer detection problem, we define the content of the training data using an extensible dictionary. Besides imaging data provided as a registered, multi-channel 3D tensor $x\in {\mathbb{R}}^{3\times X\times Y\times Z}$, we also include 3D binary region masks $𝒴\in {\mathbb{R}}^{R\times X\times Y\times Z}$ representing each of the R regions where histopathology data is included (lesions and sextants). Crucially, for this work we encode the histopathology data using a $z\in {\mathbb{Z}}^{R\times 2}$ matrix, representing a *supervision signal* {0,1,2} and a maximum *ISUP grade group* (0-negative, 1-5) for each region. The supervision signal is used to dynamically select learning objectives applicable to the type of histopathology groundtruth on a region-by-region basis inside our UCNet model, enabling large heterogeneity in the types of exams that can be included for training both within and across prostate cancer research sites. This dynamic selection is clarified in Section 2.2 when describing UCNet.

#### Federated Toolkit (FLtools)

Our lightweight FLtools library includes several components that represent baseline implementations of various federated routines (structured to enable compatibility with Nvidia’s NVFlare (13) and Flower (14), but which are crucially reusable across models and FL experiments. These include:

- FLComponents - baseline implementations of training, aggregation, and serialization strategies that utilize the get_objects interface. This decouples the model implementation from backend implementations, such as those based on Nvidia’s NVFlare or Flower, enabling reuse of all essential federated components across models and experiments.
- FLTrainer - A reusable module extending NVFlare’s “Trainer” component using the get_objects interface to perform training on the clients.
- FLAggregator - A reusable module that extends NVFlare’s “Aggregator” component, responsible for (a) validating the gradient contribution from each clients, and (b) aggregating gradient averaging via FLUtils.
- FLSharableGenerator - A reusable module that extends NVFlare’s “Sharable Generator” component and converts a model to a sharable, a dictionary of weights and gradients that can be transmitted between the server and clients.
- FLModelPersistor - A reusable model that extends “ModelPersistor”, responsible for saving and loading of the checkpoint file on the server.

As an example, Nvidia’s NVFlare environment includes “Bring Your Own Trainer” (BYOT) and “Bring Your Own Component” (BYOC) modes that enables users to develop their own FL components, e.g. to extract, aggregate, serialize, and transfer model weights and gradients between clients, using high-level APIs separated from the deep learning model architecture and data-specific code, such as pre-processing or model execution. The FLtools NVFlare backend includes baseline implementations of these components as a light-weight library that can be imported into existing research repositories, and which crucially utilizes the get_objects interface, separating local model development and FL deployment. Furthermore, FLtools includes a simulation tool FLsim that enables researchers to test FL deployment on one or more systems outside of NVidia’s federated environment (Table 3).

**Fig. 4.**
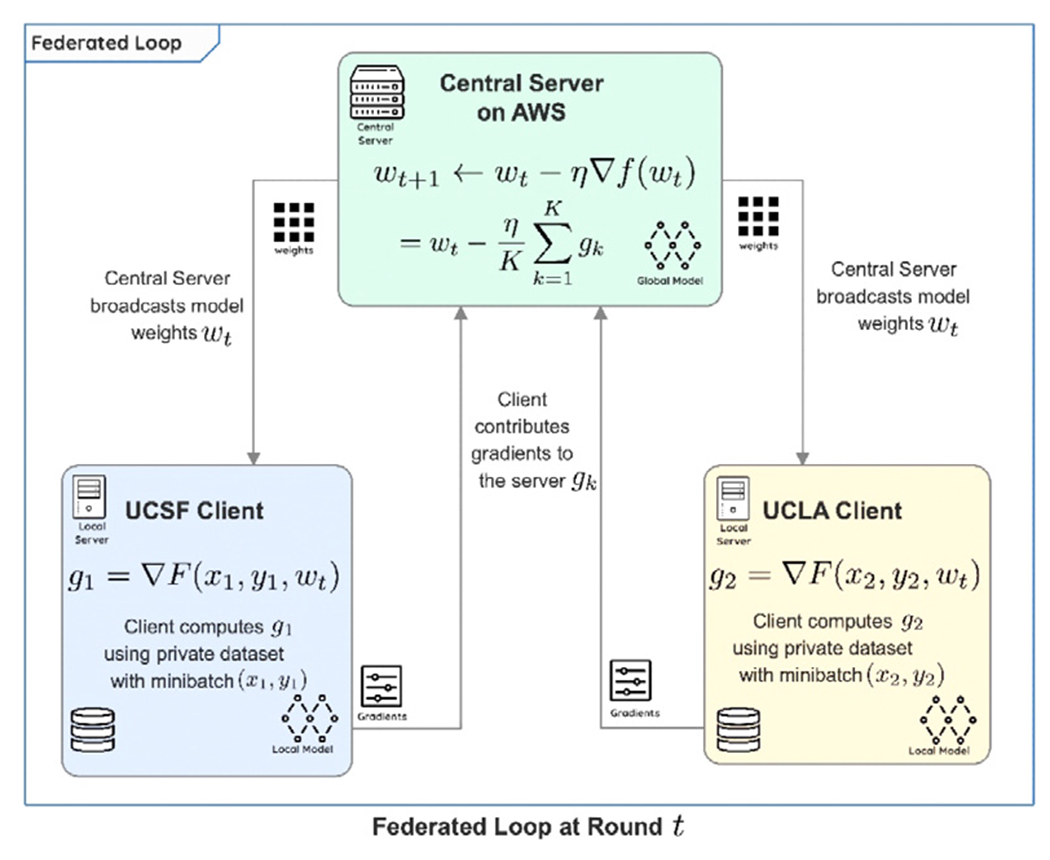
FL topology for FedSGD. (Color version of figure is available online.)

**Fig. 5.**
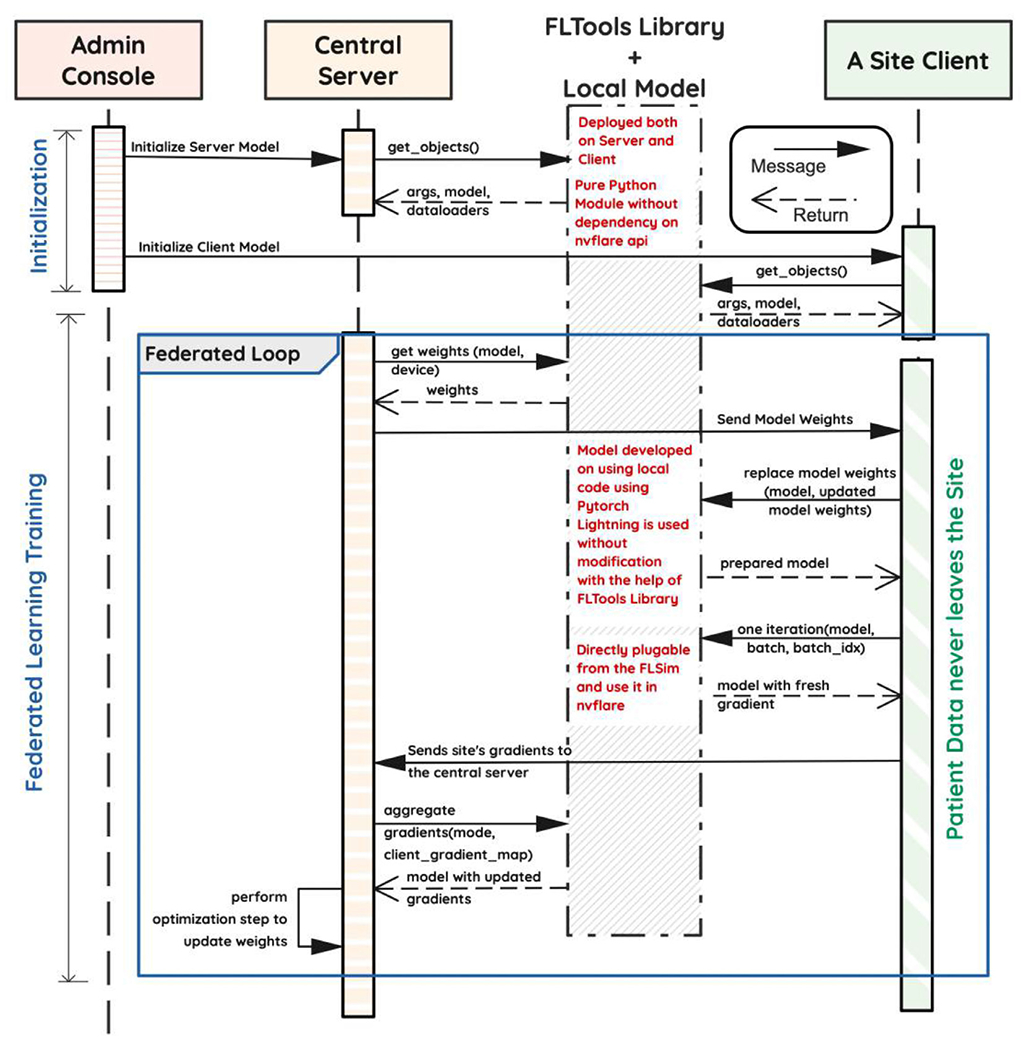
Modular federated system architecture. (Color version of figure is available online.)

Figure 5 depicts a sequence diagram elucidating the communication between various entities present in the federated topology implemented for FedSGD (Figure 4). An important feature of FLtools is the clear separation between the engineer’s duties responsible for the machine learning operations (MLOps) team and the researcher developing the model. We found that the autonomy of the respective experts to work in separate areas and integrate via a programming contract fosters an environment of collaboration, conducive to FL success.

**Fig. 6.**
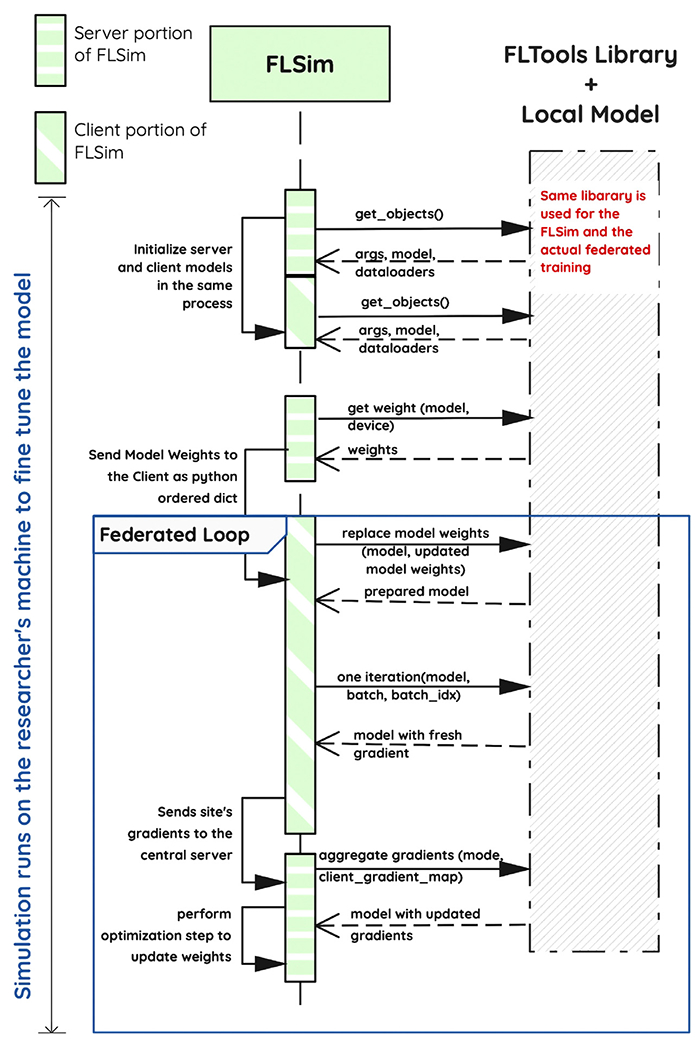
FLSim Local Federated Simulation. (Color version of figure is available online.)

Figure 6 shows how FLsim simulates the federated loop (Figure 5) on the local machine of the researcher. Since FLSim is decoupled from NVflare, the researcher is free to use existing debug tools (e.g. pdb) to fine-tune model or computational hyperparameters (e.g. batch size) prior to running the federated training via NVFlare.

#### Model Training and Implementation Details

For the FL experiments in this paper, we host the central server on an Amazon Web Services (AWS) EC2 instance, and two clients that are behind institutional firewalls at UCSF and UCLA, respectively (Figure 4). The central server aggregates gradients from each client and performs a weight update with appropriate momentum terms (we are using the AdamW optimizer with FedSGD). Our FL concept involves private data, but also private metrics, so each client has no knowledge of how well the federated model is performing at other research sites. Instead, each institution’s client monitors performance on the institution’s own private validation dataset. Thus, there is no stopping criteria from the perspective of the server, and client can freely choose to select whichever checkpoint they consider to be most performant.

## RESULTS

We evaluate the performance of UCNet in two different prostate cancer detection tasks: MR-identified lesion segmentation and lesion-wise classification of clinically significant prostate cancer (CS-PCa).

Table 4 compares the test-set mean IoU of lesion segmentation for models trained locally and via FL. The locally trained models fail (IoU = 0.000) when evaluated on data from the other site. The FL models had dramatic improvements in cross-site generalization performance for the FL models, showing reasonable performance for test set data from either site.

Since our approach utilizes *private* validation sets, clients are unaware of the performance of the model at other sites and are free to select model checkpoints with the highest validation performance independently *at each site*. This is an important point that leads to two versions of the final federated model (UCSF-FL and UCLA-FL), as each site may select different checkpoints as “best” according to their own withheld validation set.

Table 5 compares the test-set overall (class-balanced) binary lesion classification accuracy, sensitivity, and specificity for models trained locally and via FL, again indicating dramatic improvements in cross-site generalization performance for the FL models. We report occurrence-normalized (class-balanced) overall accuracy since this provides the clearest indication of a model’s generalization performance on screening populations, which is in contrast to other recent prostate cancer MRI based work that have reported results from cross-validation performance using model sensitivity and specificity (8,9,22). For this metric, lesion-wise prostate cancer classification performance is measured for each model on the population level by computing an occurrence-normalized confusion matrix and averaging the diagonal to represent the class-balanced overall accuracy. Since the region classifier in the UCNet model is not a threshold-based classifier, we do not compute a receiver operating characteristic (ROC) curve that is optimized for sensitivity and specificity using the validation set. Instead, we pick the checkpoint with the best overall accuracy on the validation set, and use this operating point for test set evaluation.

In particular, in Table 5 we can see that local models trained at each institution performed well on each institution’s private test set, but the performance on the set from the other institution was much lower, i.e. indicating poor generalization performance. UCLA-local model showed 47.9% accuracy on UCSF data, which is 30% lower than UCSF-local model performance. The result of UCLA model evaluation on UCSF data showed high TNR (0.93) and low TPR (0.03), which means low usability of the model on data in another institution. UCSF-local model showed slightly higher accuracy of 53.3% on the UCLA data, which was still 23% lower than UCLA local model result. Although the best FL model was chosen on each site separately based on the local validation performance, both FL models succeeded in generalization. UCLA-FL model increased the UCSF data classification accuracy by 14.8% with corresponding increase in TNR and TPR, which made the model usable in practice by another institution. The same result achieved UCSF-FL model, which increased UCLA local data performance accuracy by 9.5% with increase in TNR and TPR. Furthermore, the performance of the FL models on has only a small decreases in performance on local data.

**Fig. 7.**
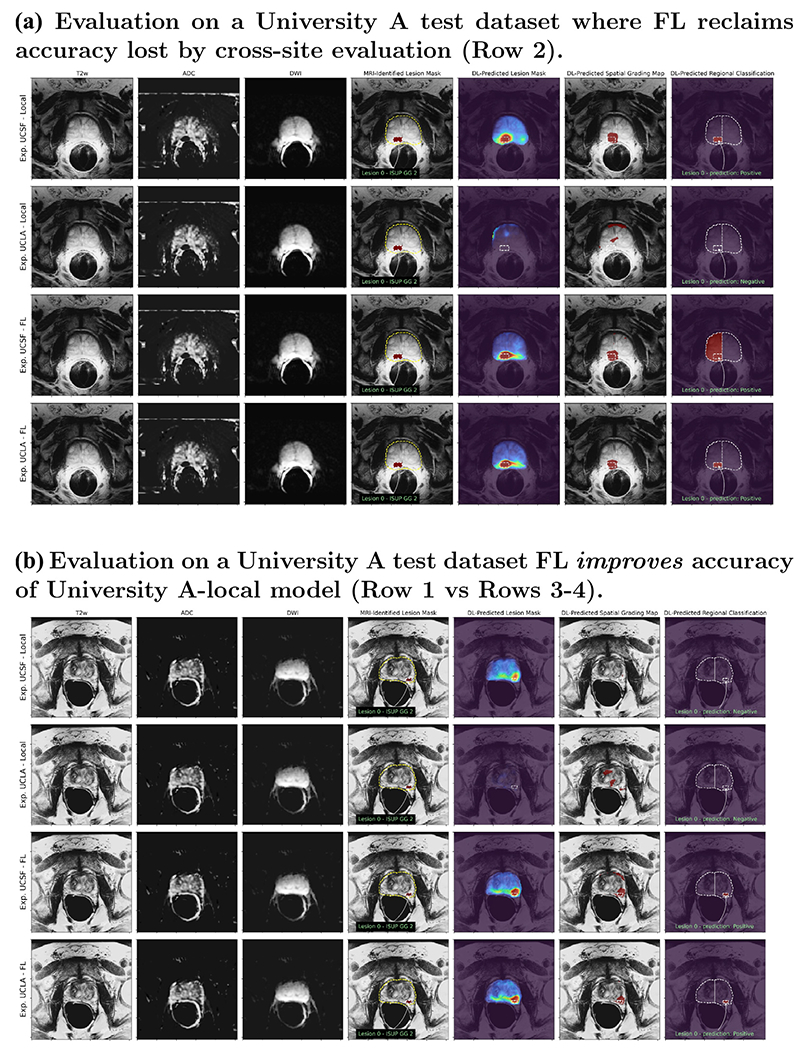
Evaluation of UCNet models on example UCSF dataset. (A) depicts a transverse slice of an exam with a MRI-identified ISUP GG 2 lesion where the UCLA-local model performs poorly, but both federated models (Rows 3-4) achieves the same level of accuracy as UCSF-local model. (B) depicts a transverse slice of an exam with a MRI-identified ISUP GG 2 lesion where both local models perform poorly, but federated models correctly classify this lesion as CS-PCa. Notably, for both (A-B), federated checkpoint chosen by UCLA stopping criteria (Row 3) performs better than the checkpoint chosen by UCSF (Row 4), highlighting that neither site has sufficient data to generalize well on their own. (Color version of figure is available online.)

**Fig. 8.**
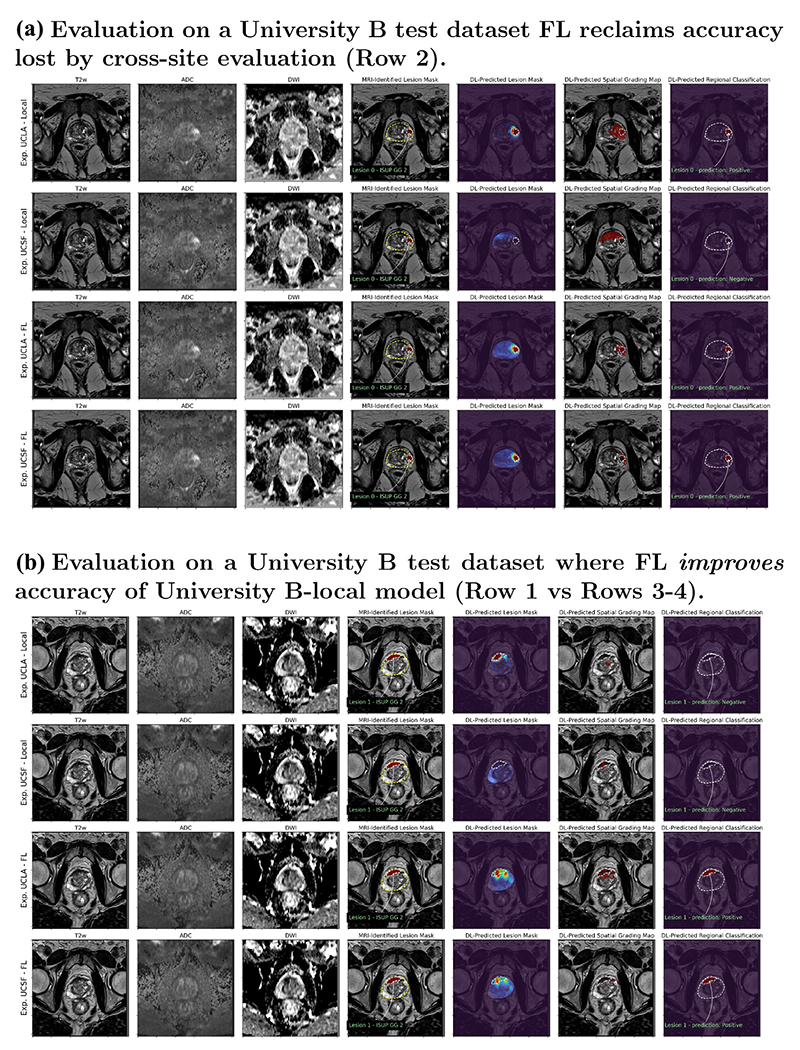
Evaluation of UCNet models on example UCLA datasets. (A) depicts a transverse slice of an exam with a MRI-identified ISUP GG 2 lesion where UCSF-local model performs poorly, but both federated models (Rows 3-4) achieves the same level of accuracy as UCLA-local model. (B) depicts a transverse slice of an exam with a MRI-identified ISUP GG 2 lesion where both local models performs poorly, but federated models perform well.

Herein, we focus primarily on classification performance of the model, so IoU performance is only reported to provide a measure of how well the model localizes areas of interest in each exam. As indicated by Figures 7 and 8, the cancer region tends to be overestimated by the model in most exams. The false positive rate (Type-I error) for classification can be inferred from the Table 5 as 1 - TNR (true negative rate); this indicates a 26-33% initially on local models and data trained at each site, and with 7-45% when the local models were initially exchanged. Note that the low false positive percentage is not indicative of the overall model accuracy, as the true positive rate was also low (3-51%) when models were initially exchanged, resulting in poor overall performance. For example, the 7% FPR with 3% TPR indicates that the model tends to predict mostly ‘Negative‘ disease. However, the results in Table 5 further demonstrate a positive result for the federated learning experiment, indicating a boost to 35-40% for federated checkpoints picked and evaluated on data from the same institution, and 43-53% for federated checkpoints evaluated on data from the other institution. Combined with improvements in reducing the false negative rate (Type-II error), the federated experiment demonstrates greater generalization accuracy in terms of class-balanced accuracy (average of the true negative and true positive rates).

In Figures 7 and 8 we illustrate the performance through sample results of local and FL models from both institutions evaluated on UCSF and UCLA test sets, respectively. These figures show the input images and MRI-identified lesion masks along with the DL-predicted results from UCNet models. These include lesion masks that aim to predict and highlight the lesion location, spatial grading map that aims for a voxel-wise prediction of CS-PCa, and region-wise classifications of CS-PCa for each sextant and lesion.

As mentioned above, local UCSF and UCLA models performed well on data from their own site, but did not generalize well to data from the other site, which is shown in Row 2 of Figure 7.A and Figure 8.A. In these examples, the MRI-identified lesion is not identified in the DL-predicted lesion mask, spatial grading map, or regional classification. The last two rows of the figures show that in these examples the FL models are able to reclaim accuracy loss for improved generalization. The DL-predicted lesion mask in particular clearly highlights the MRI-identified lesion with both FL models and on test data from both sites.

While overall FL models evaluated on the same sites resulted in a slight accuracy drop, we observed that in some cases FL improved the performance of the local model as shown in Figure 7.B and Figure 8.B. In these cases, the local models were able to identify the lesion location, indicated by the intensity of the DL-predicted lesion mask, but failed to correctly classify this CS-PCa (ISUP GG 2) in these patients. Interestingly, the FL models both were able to correctly identify these lesions as CS-PCa (Rows 3-4) in the spatial grading map and regional classification.

## DISCUSSION

The primary goals of federated learning are to increase the absolute performance of models but also to improve the generalization capability of models across institutions. To this end, we challenged our FL system and deep learning model by presenting highly heterogeneous MRI data, patient distributions, and groundtruth annotations (both within and across institutions). Our bespoke architecture, UCNet, was able to handle this task, and resulted in good performance in both local and federated training. The resulting models, chosen on each site independently based on the local validation performance, gained the benefit of having learned from each of the private datasets without ever needing to transfer, pool, or homogenize data at a single location. Both FL checkpoints showed desirable generalization performance, and resulted in higher accuracy evaluated on the local datasets from the opposite institutions, while neither site had sufficient data to generalize well on their own. The clinical impact of federated CS-PCa detection models is improved generalization accuracy and physician confidence in deployed models.

One design choice that should be revisited is the stopping criteria for choosing checkpoints independently on private institutional validation data. Although private validation increases privacy and incentives institutions to participate in FL to improve performance on their own data, we believe a better methodology may be to monitor global validation accuracy since the accuracy on the other institution’s dataset may give a better indicator of performance on out-of-distribution data, the common pitfall all of deep learning models. This is evident from the deviation between within-site performance on validation and test sets, which was especially bad for UCLA data with −15.4% classification accuracy and −45% lesion segmentation IoU.

In terms of practical FL implementation, the presented FedSGD technique implemented in FLComponents achieves *O*(1) memory complexity with respect to the number of clients, and thus is highly scalable for training with hundreds of research sites. However, one practical issue with FedSGD is that it has a relatively high time and communication complexity O(n), since it must wait until every client responds before updating the global model *at every iteration*. In this respect, FedAvg may be a more desirable aggregation strategy, although the training dynamics have not yet been explored with UCNet and prostate MRI. One option to reduce the complexity constants for FedSGD may be to implement asynchronous peer-to-peer distribution of gradients and local update of weights, with the central server only acting to globally update momentum terms. We leave this as future work for the FLtools library.

In addition to prostate MRI other medical imaging applications that would directly benefit from this approach include classification of less common cancers, e.g. brain tumors, kidney cancer and pancreatic cancer, where the ability to utilize data from multiple sites and all sources of histology groundtruth can significantly increase dataset sizes to potentially boost overall detection accuracy and/or generalization (24). The presented FL design pattern is especially useful for data-starved deep learning problems that are challenging enough to necessitate the use of non-standard architectures or loss functions, where its desirable to expand dataset size and diversity while retaining model, dataloader, and learning algorithm mutability (12,25)

More broadly, we hope that this work will encourage collaboration and data-sharing across institutions, enabling us to tackle the next phase of model training in radiology. Considerations here include increasing input data diversity label granularity diversity, and finding/demographic diversity. In this way, we believe that federated learning has an important role to play in addressing health equity issues by improving representation of underserved populations (26), such as for Black prostate cancer patients (27).

## CONCLUSION

We develop and open-source a federated learning toolkit FLtools that can be combined with the powerful NVFlare and Flower backends to provide an extensible and reusable federated components for researchers to re-use with custom deep learning models and workflows, or with heterogeneous datasets that can vary in specification across clients in a federation. We successfully applied this design pattern to train a custom UCNet deep learning model we developed to handle diverse prostate MRI and associated radiological and histopathology annotations using two large datasets of 1800+ exams. Results indicate between 9.50-14.8% improvement in generalization performance of lesion classification, and nearly 100% improvements in generalization performance of lesion segmentation. To improve absolute performance of models and realize the full potential of federated learning, incorporation of additional multi-institution data is required. The tools and approach presented will readily support these efforts for improved assessment of prostate cancer based on MRI.

## Figures and Tables

**TABLE 1.** Number of exams as a function of ISUP grade group.

	UCSF-Train	UCSF-Val	UCSF-Test	UCLA-Train	UCLA-Val	UCLA-Test
max ISUP 0	92	17	24	196	26	43
max ISUP 1	222	27	73	172	24	31
max ISUP 2	228	30	61	197	27	40
max ISUP 3-5	137	22	40	172	24	34
Totals	679	96	198	737	101	148

**TABLE 2.** Cohort demographics for the prostate MRI datasets from UCSF and UCLA used in this study.

	UCSF	UCLA
Age (years)	66.6 ± 7.2	64.5 ± 7.4
PSA Density (ng/ml^2^)	0.18 ± 0.21	0.14 ± 0.27
White	750 (77.1%)	625 (63.4%)
Asian	60 (6.2%)	55 (5.6%)
Black	34 (3.5%)	37 (3.7%)
Other/unknown/declined	129 (13.2%)	269 (27.3%)
Endorectal Coil	813 (83.6%)	17 (1.7%)

**TABLE 3.** Comparison of Features in Different Federated Learning Frameworks.

	NVFlare 1.0 + Clara 4.0	NVFlare 1.0 + BYOT + BYOC	NVFlare 2.0 Custom FLComponents	Our System (NVFlare + FLTools)
Use the Clara Models for Training	✓	✓	✓	✓
Bring your model without code modification	✘	✘	✘	✓
Use custom dataloader	✘	✓	✓	✓
Check FL loop simulation without NVFlare API	✘	✘	✘	✓
Fix training issues using python debugger	✘	✘	✓(poc mode)	✓
Custom Checkpointing and Transfer Learning	✘	✓	✓	✓
Using the local logging without modification	✘	✘	✘	✓

**TABLE 4.** Mean lesion segmentation intersection-over-union (IoU) on test sets.

Model Test Set	UCSF	UCLA
UCSF-local	0.134	0.000
UCSF-FL	0.120	0.105
UCLA-local	0.000	0.153
UCLA-FL	0.111	0.063

**TABLE 5.** Region-wise Lesion Binary Classification Accuracy. Bracketed numbers indicate TNR and TPR, respectively.

Model Test Set	UCSF	UCLA
UCSF-local	68.0% [0.74, 0.63]	53.3% [0.55, 0.51]
UCSF-FL	67.9% [0.76, 0.60]	62.8% [0.69, 0.57]
	↓ 0.01%	↑ 9.5%
UCLA-local	47.9% [0.93, 0.03]	69.5% [0.67, 0.73]
UCLA-FL	62.7% [0.78, 0.47]	66.8% [0.74, 0.65]
	↑ 14.8%	↓ 2.7%
